# *CTLA4* Polymorphisms and De Novo Malignancy Risk after Renal Transplantation in Chinese Recipients

**DOI:** 10.1155/2015/986780

**Published:** 2015-01-15

**Authors:** Yi-feng Guo, Jian-xin Qiu, Fang Guo, Yong Liu, Ming-hua Shang

**Affiliations:** ^1^Organ Transplantation Center, Shanghai First People's Hospital, School of Medicine, Shanghai Jiao Tong University, 100 Haining Road, Shanghai 200080, China; ^2^Key Laboratory of Systems Biology, Shanghai Advanced Research Institute (SARI), Chinese Academy of Sciences, 99 Haike Road, Shanghai 201210, China; ^3^Department of Nephrology, Shanghai First People's Hospital, School of Medicine, Shanghai Jiao Tong University, Shanghai 200080, China

## Abstract

Genetic polymorphisms in cytotoxic T lymphocyte-associated antigen 4 (*CTLA4*) play an influential role in graft rejection and the long-term clinical outcome of organ transplantation. We investigated the association of five *CTLA4* single-nucleotide polymorphisms (SNPs) (rs733618 C/T, rs4553808 A/G, rs5742909 C/T, rs231775 A/G, and rs3087243 G/A) with de novo malignancy in 1463 Chinese renal transplantation (RT) recipients who underwent a 192-month follow-up. Multivariate analyses revealed that recipient rs231775 genotype is significantly associated with tumorigenesis (*P* = 0.012). Multiplicative interaction between rs231775 AA and possible risk factors of malignancy revealed two significant results: rs231775 AA × primary diseases and rs231775 AA × number of HLA-mismatch. The frequency of haplotype TACAG was significantly higher in the tumor group (17.07%) than that in the nontumor group (1.53%). In addition, aristolochic acid nephropathy (*P* = 0.003) and the time of discovery of tumor (*P* = 0.000) also were independently associated with tumorigenesis. Our data show that the *CTLA4* genotype rs231775 AA may be one of risk factors for the development of malignancy and haplotype TACAG was susceptible haplotype in Chinese kidney transplant recipients.

## 1. Introduction

Recipients with malignant tumors after renal transplantation are one of important factors affecting the long-term survival and are one of the first four most common causes of death following cardiovascular disease, infection, and liver failure [1]. Risk of malignancy after transplantation increases with the extension of time [2]. Due to own humoral and cellular immune defects, there was high risk of malignancy in uremic patients. Kidney transplantation has become effective alternative treatment methods of uremia. The inventions of new immunosuppressive agents reduced the incidence of acute rejection and improved short-term graft survival rate, but long-term survival has not been significantly improved. With higher potency, a more direct role in the continuous advent of new immunosuppressive agents, kidney transplant short-term effect has been greatly improved, while, with the number of elderly patients receiving transplants increasing, the risk of cancer after transplantation also will increase.

Cytotoxic T lymphocyte-associated antigen 4 (*CTLA4*) is a key element in the immune system that induces immune tolerance and is one of the critical negative regulators of the T cell-mediated immune response [3]. It is also expressed constitutively on the surface of regulatory T cells (Tregs) and is detectable on approximately 50% of Tregs; it is only found on <1% of naive helper T cells [4]. CTLA4 ligation on Tregs results in a significant decrease in the presentation capacity of antigen-presenting cells and effector T cell downregulation in mice [5]. As* CTLA4* plays an important role in the downregulation of the immune response, the single-nucleotide polymorphisms (SNPs) of the* CTLA4* gene +49 A/G (rs231775) and +6230 G/A (rs3087243) are associated with autoimmune diseases [6, 7] and play an influential role in graft rejection and the long-term clinical outcome of organ transplantation [8–10].

Recently, some studies discover that single nucleotide polymorphisms (SNPs) of the* CTLA4* gene have been implicated in susceptibility to different cancer in different ethnic populations [11–16]. The role of* CTLA4* SNPs in T cell mediated immunity after transplantation on the de novo malignancy is unknown. Therefore, this study was designed to investigate the associations between five* CTLA4* SNPs (rs733618 C/T, rs4553808 A/G, rs5742909 C/T, rs231775 A/G, and rs3087243 G/A) and malignancy in Chinese renal transplantation recipients.

## 2. Materials and Methods

### 2.1. Diagnostic Criteria and Methods

Inclusion criteria were as follows: ① preoperative examination showing no malignancy, ② successful kidney transplantation surgery, ③ long-term use of immunosuppressive agents after transplantation, ④ and experimental methods for the treatment of patients with informed consent and approval by the hospital ethics committee.

People having the following conditions were excluded from the study: ① preoperative history of cancer, ② dialysis being as substitute for graft loss during follow-up, ③ transplanted graft being removed after renal transplantation, ④ and being disabled or no long-term use of immunosuppressive agents following renal transplantation.

### 2.2. Patients

In 1463 cases, the number of recipients who met the study criteria was 464 cases (288 men and 176 women) in the Shanghai Organ Transplantation Center by December 2013. 53 recipients had tumors, and 411 cases had no tumors. Of the 53 patients with tumors, 20 cases presented with urinary system tumors (renal cancer: 12 cases, ureteral carcinoma: 2 cases, bladder cancer: 5 cases, and prostate cancer: 1 case), 18 with gastrointestinal tumors (liver cancer: 10 cases, esophageal carcinoma: 1 case, gastric cancer: 1 case, and colorectal cancer: 6 cases), and 12 with other kinds (lung cancer: 4 cases, nasopharyngeal carcinoma: 2 cases, laryngeal cancer: 1 case, tongue cancer: 1 case, breast cancer: 5 cases, uterine cancer: 1 case, and brain cancer: 1 case). The mean age of the patients (age at time of renal transplantation) included in the study was 40.24 ± 10.02 years. All of the recipients were blood group-matched with their donors and were tested for the panel-reactive antibody and HLA-A-B-DR matching.

Patients gave written informed consent to the collection and storage of blood, isolation of DNA, and determination of gene polymorphisms. The study protocol was conducted in accordance with the Declaration of Helsinki and its amendments and was approved by the Ethics Committee of Shanghai Jiao Tong University. Each patient underwent a 192-month follow-up observation through which clinical information was provided by means of clinical observation, medical records, and outpatient or telephone follow-up visits.

### 2.3. Immunosuppression Protocol

Mycophenolate mofetil (MMF) 1.0 was given as a premedicant. Antibody induction therapy (including monoclonal antibody, antithymocyte globulin, and antilymphocyte globulin) was used. Intravenous infusion of 500 mg/d of methylprednisolone was applied during the procedure through 2 days after the operation. The dose was then decreased to 360 mg, 180 mg, 80 mg, and 40 mg each subsequent day, followed by prednisone (15–20 mg/d) as a maintenance therapy. Triple therapy with cyclosporine A (CsA)/tacrolimus (TAC), MMF, and prednisone was administered beginning on the third day after the operation. The dosage of MMF was 1.0–1.5 g/d with a weight of 60 kg as the critical value. CsA and TAC were started at doses of 8 mg/kg/d and 0.2 mg/kg/d, respectively, and then adjusted according to the plasma concentrations and the serum creatinine concentrations.

The diagnostic criteria of AR were based on the comprehensive elevation of histological and clinical symptoms and their alleviation by antirejection therapy and graft biopsy. The clinical symptoms examined were hypourocrinia, fever, weight gain, pain in the transplanted kidney, elevated blood pressure, increased serum creatinine (to 25% above baseline), urine protein, and the resistance index. The Banff 97 working classification for renal allograft pathology (modified) [17] was used as the pathological rejection criteria.

### 2.4. Sample Collection and Polymorphism Genotyping

A total of 464 patients were included in this study. Peripheral blood samples (3 mL) were collected, the DNA was extracted, and the SNPs of* CTLA4* were genotyped using polymerase chain reaction (PCR) and direct sequencing. For the primers and annealing temperatures (ATs) employed for rs733618 C/T, rs4553808 A/G, rs5742909 C/T, rs231775 A/G, and rs3087243 G/A, refer to [18].

### 2.5. Statistical Analysis

Comparisons of clinical characteristics between patients with tumor and nontumor were analyzed by the Pearson *χ* ^2^ test and an independent-sample test. We assessed the Hardy-Weinberg equilibrium (HWE) for both tumor and nontumor using the *χ* ^2^ test. For linkage disequilibrium (LD), Haploview version 4.2 software was used. G^*^Power 3.1.9.2 was used in power calculation containing the whole samples and every allele distribution [19]. Genotype associations were analyzed using a dominant model (minor-allele homozygotes plus heterozygotes versus major-allele homozygotes) and a recessive model (minor-allele homozygotes versus heterozygotes plus major-allele homozygotes). The allelic frequencies were counted in a single strand of measured DNA. The differences in the genotype distributions between groups were analyzed by the *χ* ^2^ test or Fisher's exact test. Associations of the* CTLA4* SNPs with the time of discovery of tumor in patients were analyzed by the Kaplan-Meier test. Multivariate analyses were used to analyze several risk factors, including age at time of transplantation, gender, primary diseases, dialysis time, number of HLA-mismatches, antibody induction therapy, acute rejection, blood transfusion,* CTLA4* SNPs, and the time of discovery of tumor. Four SNPs (rs733618, rs4553808, rs5742909, and rs231775) have been previously reported as haplotype-tagged SNPs at the CTLA4 locus [20]. We explored the haplotype association for 5 SNPs using Haploview version 4.2. Correction for multiple testing was carried out using the Bonferroni method. *P* values for gene-environment interaction and gene-gene interaction were calculated using the multiplicative interaction term in SPSS software. Statistical analysis was performed with SPSS (Statistical Package for the Social Sciences) version 11.5 software (SPSS Inc., Chicago, IL, USA). All statistical tests were two-sided, and statistical significance was set at *P* < 0.05.

## 3. Results

### 3.1. Baseline Characteristics of 464 Renal Transplant Recipients

**Figure 1:**
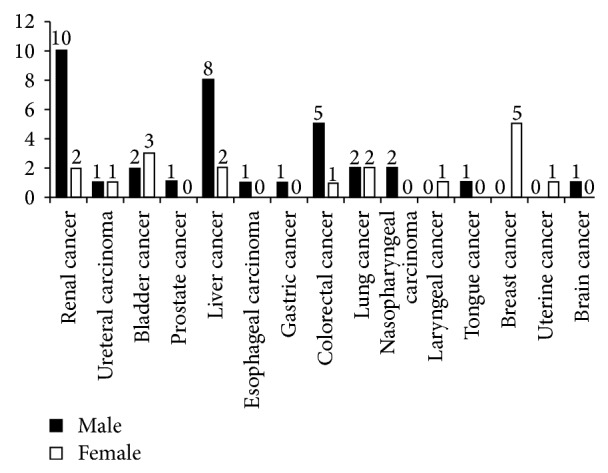
Types of tumors discovered in recipients following renal transplantation.

The total number of patients was 1463; 464 cases were included in the study (including 53 recipients with tumors) in observation period (192 months), with 288 male and 176 female cases. A total of 3.62% recipients (53/1463) had tumor. Baseline characteristics of 464 renal transplant recipients and types of tumor were listed in Table 1. No significant differences in age, sex, pretransplant dialysis time, human leukocyte antigen mismatches, real transplantation, blood transfusion, antibody induction therapy, immunosuppressant regimen, or acute rejection (AR) were found between patients with tumor and those without. In primary diseases, except for chronic glomerulonephritis, polycystic kidney, and pyelonephritis, percent of patients with aristolochic acid nephropathy was different between tumor group (5.66%) and nontumor group (0.73%) (*P* = 0.003). Fifty-three patients were diagnosed as tumor patients within the 192 months after operation; the kinds of tumors were listed in Figure 1.

*Associations between the CTLA4 SNPs and Tumor*. All polymorphisms were in Hardy-Weinberg equilibrium (Supplementary Table  1 available online at http://dx.doi.org/10.1155/2015/986780). Using Haploview version 4.2 software, the five loci were found to be in linkage disequilibrium (LD) (*D*′ = 0.920–1.000). The power calculation was made between tumor group and nontumor group, and power value was 0.964. To CTLA4 five loci allele, the power was calculated, respectively. Except for rs4553808 G (power = 0.696), rs5742909 C (power = 0.658), and rs3087243 A (power = 0.519), the rest of allele powers were in high value (0.898–0.998). Regarding the genotype distribution of the* CTLA4* polymorphisms, no statistical differences for rs733618, rs4553808, rs5742909, or rs3087243 were found between patients with tumor and those without. However, the frequency of the rs231775 AA genotype in recipients with tumor was higher (48.89%) than in those recipients without tumor (37.73%) (*P* = 0.012, OR = 1.579, and 95% CI = 1.019–2.447), but when corrected, *P* value > 0.05 (Bonferroni-adjusted *P* = 0.06) (Supplementary Table  2).

No differences in the determined allelic frequencies of rs733618, rs4553808, rs5742909, or rs3087243 were found between tumor and nontumor recipients. The frequency of the allelic rs231775 A in recipients with tumor was higher (%) than in those recipients without tumor (%) (*P* = 0.011, OR = 0.594, and 95% CI = 0.396–0.892), but when corrected, *P* value > 0.05 (Bonferroni-adjusted *P* = 0.055) (Supplementary Table  3).

Kaplan-Meier analysis was used to examine the relationships between* CTLA4* SNPs and tumor; no statistical differences for rs733618, rs4553808, rs5742909, or rs3087243 existed between tumor and nontumor recipients. A significant difference (*P* = 0.014) was found between patients bearing the rs231775AA genotype and those with the GG+AG genotypes using the log-rank test. A significant association was found between the rs231775 genotype and the time of discovery of tumor in recipients (Supplementary Figure  1).

To further examine the associations of tumor with these variables, multivariate analyses were carried out with the variables age at time of transplant, gender, primary disease, pretransplant dialysis time, HLA-mismatch, antibody induction therapy, immunosuppressant regimen, blood transfusion, renal transplantation, acute rejection, rs231775 genotype, and the time of discovery of tumor.

Multivariate analyses revealed that age at time of transplant, gender, primary disease (except for aristolochic acid nephropathy), pretransplant dialysis time, HLA-mismatch, renal transplantation, immunosuppressive regimen, blood transfusion, and acute rejection were not independent of tumor; however, the analyses showed that three risk factors, recipient rs231775 genotype (*P* = 0.012), aristolochic acid nephropathy (*P* = 0.003), and the time of discovery of tumor (*P* = 0.000), were independently associated with tumor (Supplementary Table  4).

### 3.2. Multiplicative Interaction among Possible Risk Factors of Malignancy

The multiplicative interaction was tested in age at transplantation, gender, primary diseases, dialysis time, number of HLA-mismatch, antibody induction therapy, and acute rejection. Age at transplantation × sex: *P*_interaction_ = 0.187; age at transplantation × primary diseases: *P*_interaction_ = 0.053; Age at transplantation × dialysis time: *P*_interaction_ = 0.612; age at transplantation × number of HLA-mismatch: *P*_interaction_ = 0.966; age at transplantation × antibody induction therapy: *P*_interaction_ = 0.001; age at transplantation × AR: *P*_interaction_ = 0.080; sex × primary diseases: *P*_interaction_ = 0.766; sex × dialysis time: *P*_interaction_ = 0.560; sex × number of HLA-mismatch: *P*_interaction_ = 0.886; sex × antibody induction therapy: *P*_interaction_ = 0.838; sex × acute rejection: *P*_interaction_ = 0.590; primary diseases × dialysis time: *P*_interaction_ = 0.043; primary diseases × number of HLA-mismatch: *P*_interaction_ = 0.815; primary diseases × antibody induction therapy: *P*_interaction_ = 0.002; primary diseases × acute rejection: *P*_interaction_ = 0.094; dialysis time × number of HLA-mismatch: *P*_interaction_ = 0.055; dialysis time × antibody induction therapy: *P*_interaction_ = 0.275; dialysis time × acute rejection: *P*_interaction_ = 0.057; number of HLA-mismatch × antibody induction therapy: *P*_interaction_ = 0.281; number of HLA-mismatch × acute rejection: *P*_interaction_ = 0.981; antibody induction therapy × acute rejection: *P*_interaction_ = 0.282 (Table 2).

### 3.3. Multiplicative Interaction between rs231775 AA and Possible Risk Factors of Malignancy

The multiplicative interaction was tested in rs231775 AA with possible risk factors, including age at transplantation, dialysis time, gender, primary diseases, antibody induction therapy, number of HLA-mismatch, and acute rejection. rs231775 AA × age at transplantation: *P*_interaction_ = 0.712; rs231775 AA × dialysis time: *P*_interaction_ = 0.078; rs231775 AA × gender: *P*_interaction_ = 0.306; rs231775 AA × primary diseases: *P*_interaction_ = 0.021; rs231775 AA × antibody induction therapy: *P*_interaction_ = 0.364; rs231775 AA × number of HLA-mismatch: *P*_interaction_ = 0.006; rs231775 AA × acute rejection: *P*_interaction_ = 0.331 (Table 3).

### 3.4. The Association of CTLA4 Haplotype with Tumor

**Figure 2:**
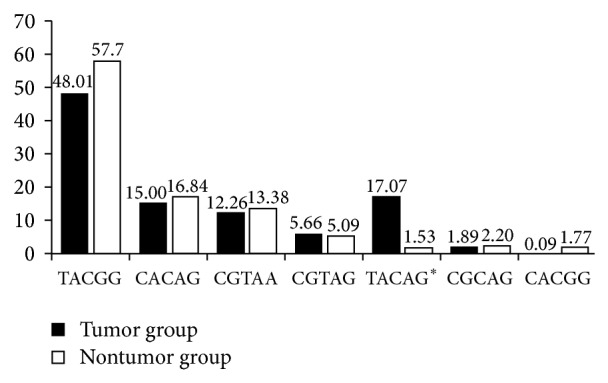
The distribution of haplotypes in five loci of* CTLA4* between the recipients with tumor and with nontumor. Black bar: haplotypes frequency of tumor group; white bar: haplotypes frequency of nontumor group. ^∗^ *P* value < 0.0000 and permutation *P* value < 0.0000.

No differences in the frequencies of six haplotypes (TACGG, CACAG, CGTAA, CGTAG, CGCAG, and CACGG) covering the 5 SNPs existed between the tumor and nontumor recipients; the frequency of haplotype TACAG was significantly higher in the tumor group (17.07%) than in the nontumor group (1.53%) (*P* = 0.0000 and permutation *P* value = 0.0000) (Figure 2).

## 4. Discussion

The* CTLA4* SNPs have been implicated in susceptibility to various cancers in different ethnic populations. These SNPs, mainly including rs231775 A/G, rs5742909 C/T, rs3087243 G/A, rs733618 C/T, and rs4553808 A/G, were involved in various tumors such as breast cancer, lung cancer, and pancreatic cancer [11–16]. Our study was designed to investigate the associations between five* CTLA4* SNPs (rs733618 C/T, rs4553808 A/G, rs5742909 C/T, rs231775 A/G, and rs3087243 G/A) and de novo malignancy in Chinese renal transplantation recipients.

Our study revealed that the frequency of recipients carrying the rs231775 AA genotype and the rs231775 A allele in the tumor cohort was higher than that in the nontumor group (26.42% and 51.89%; *P* = 0.012 and *P* = 0.011, resp.). Conversely, the frequency of recipients carrying the rs231775 GG genotype and rs231775 G allele in the nontumor cohort was relatively higher (35.28% and 60.95%). These results are consistent with the previously reported finding in which the rs231775 G allele could mitigate the negative effect of* CTLA4* on T cell-mediated immune responses [21], also consistent with the study. However, the statistical significance between groups did not hold after correction for multiple testing. This may simply be due to the sample size and, hence, lack of power to detect an association.

The frequency of the G allele at the* CTLA4* +49 (rs231775) locus is much higher in the Chinese population than in other populations [22]. This may indicate an even more significant role of this genetic bias. In addition, using log-rank analysis, we discovered that the rs231775 AA genotype was associated with the time of discovery of tumor (*P* = 0.014) (Supplementary Figure  1).

The sample size and power influence the accuracy and authenticity of statistical results indeed. In our paper, the power values in the whole sample and most alleles were higher than the three alleles including rs4553808 G, rs5742909 C, and rs3087243 A. The causes of low power value may be due to small sample size and low allele frequency. Next, we need larger sample size to come to the more definitive conclusion that the SNPs are associated with the phenotypes.

To further examine the associations of tumor with these variables, multivariate analyses were carried out for the following variables: age at time of transplant, gender, primary disease, pretransplant dialysis time, HLA-mismatch, blood transfusion, antibody induction therapy, immunosuppressant regimen, acute rejection (AR), rs231775 genotype, and the time of discovery of tumor.

Malignancies after kidney transplantation are one of the major complications of long-term immunosuppression. The occurrence of tumors has an important relationship. According to statistics, in patients suffering from malignant renal transplantation, cancer risk is 3–5 times higher than the general population of the same age, and some tumors' risk is even up to 100 times [23]. According to some literature, in the long-term survival patients after renal transplantation, malignant tumor incidence was 4%–18%, with an average of 6% [24]. The occurrence rate is 0.56%–4.2% in China, and the overall incidence rate is 1.5%, including urinary neoplasms (accounted for 30.48%, the most common tumor type after renal transplantation in China) [25]. In our paper, the occurrence rate of tumor was 3.62%, which is consistent with incidence rate of de novo malignancy after transplantation. Skin cancer and tumor lymphatic system tumors are often common, in recipients after renal transplantation in the United States and Europe, followed by urinary tract tumors [26]. In Korea and Japan, gastrointestinal tumors are more common, in which incidence rate was 40% and 57%, respectively [27, 28], while urinary tract tumors, mainly urothelial tumors, are mostly common in China (30%) [29]. In our paper, the incidence of various tumors is as follows (see Figure 1), including urinary tract tumors in 20 cases, accounting for 37.74% of the total number of tumors, followed by 18 cases of gastrointestinal cancer and 15 cases of other tumor.

The reasons for high incidence of urinary tract tumors in China may be associated with some fields. First, the high incidence can be related to the long-term use of herbs before transplantation containing aristolochic acid [30]. Second, it can be related to the fact that the original kidney transplant stops the secretion of urine, or urine secretion was minimal; the original kidney and urinary tract erosion weaken the original kidney, and the remnants of metabolites stimulate long-term sustainability in the urinary tract transitional cell epithelium, causing the cancer [31, 32]. Third, the organic material contact history, smoking history, urinary tract infections, and abuse of analgesics in patients with malignancies after kidney transplantation are risk factors for kidney tumors, and receptors existence of their own tumor recurrence is not ruled out.

Studies have pointed out that the application of antithymocyte globulin/antilymphocyte globulin or OKT3 increased posttransplant lymphoproliferative disease incidence in year 1; other immunosuppressive agents inhibit T lymphocytes, while also causing cell mutation and distortion effect. Azathioprine and mycophenolate mofetil do not increase the shift to lymphoproliferative disease incidence after planting, but the posttransplant Kaposi's sarcoma incidence was significantly increased. Tacrolimus and cyclosporine, calcineurin inhibitors, can increase the expression of transforming growth factor, thereby promoting tumor invasion, metastasis, and recurrence. Only immunosuppressant RPM may inhibit occurrence and recurrence of tumors, besides having strong immunosuppressive efficacy [33]. In our study, multivariate analysis has not showed that antibody induction therapy (including monoclonal antibody, antithymocyte globulin, and antilymphocyte globulin) was a risk factor for malignancy after transplantation. But in multiplicative interaction among malignancy and other possible factors, there was significant antibody induction therapy-primary diseases interaction (*P*_interaction_ = 0.002).

The data analysis in Marcén et al. [34] and Kauffman et al. [35] showed that age and male malignancies after kidney transplantation are a risk factor; in this group, cancer incidence rate is 3.58% for men, which is higher than women (2.57%). In our paper, the difference between men and women has not been found (*P* = 0.552).

In our study, there were 2 patients at 50 years of age, seven cases at 30–39 years, eleven cases at 40–49 years, fifteen cases at 50–59 years, and twenty cases over 60 years, suggesting that renal transplant in patients over the age of 50 may increase risk of cancer incidence. Although age at transplantation is not significantly associated with the development of tumor using multivariate analysis (*P* = 0.712), which contradicts some previous studies, we discovered that there was a significantly multiplicative age at transplantation-antibody induction therapy interaction (*P*_interaction_ = 0.001).

In 2004, Kasiske et al. [36] also believe that hemodialysis time ≥3 years after renal transplantation is a risk factor for cancer. We carried out the multivariate analysis and discovered no difference between pretransplant dialysis time ≥3 years and <3 years (*P* = 0.625), but there existed a significantly multiplicative dialysis time-primary diseases interaction (*P*_interaction_ = 0.043). Another study has shown that donors and recipients of HLA matching locus and the occurrence of cancer after transplantation, HLA-B, and two sites in a mismatch risk was 1.4 (0.5–4.1) and 5.1 (1.4–19.0) [37]. In our paper, number of HLA-mismatching was not associated with the development of tumor using multivariate analysis (*P* = 0.429), but there significantly existed a multiplicative interaction between rs231775 AA and number of HLA-mismatch (*P*_interaction_ = 0.006). So, we discovered rs231775 AA and number of HLA-mismatch may be risk factors for incidence of malignancy in gene-gene interaction. In Europe and Unites States, posttransplant malignancy risk may be associated with sun exposure [38]; incidence of skin cancer is common. In our paper, no skin cancer has been discovered yet.

Multivariate analysis showed three risk factors; besides the two risk factors which are recipient rs231775 genotype (*P* = 0.040) and the time of discovery of tumor (*P* = 0.000), aristolochic acid nephropathy was also an important risk factor (*P* = 0.003). We further discovered that there significantly was a gene-environment interaction between rs231775AA and primary diseases (*P*_interaction_ = 0.021). In China, some patients suffer from nephropathy due to using Chinese herbs containing aristolochic acid (AA) [30]. In our study, primary diseases were aristolochic acid nephropathy in 3 cases with de novo malignancy, associated with diet pills (containing aristolochic acid).

In our study, the frequency of* CTLA4* haplotype TACAG, which includes the rs231775A allele, was significantly higher in the tumor group (17.07%) than in the nontumor group (1.53%) (*P* = 0.0000). In the document on association of* CTLA4* SNP with risk of tobacco-related oral carcinoma in high-risk North Indian population [12], TACAG appeared as susceptible haplotype with TACGA and TATAG, while TACGG and CACGG appeared as protective haplotypes. In our paper, frequency of haplotypes, TACGG and CACGG, in nontumor group was higher than that in the tumor group (Figure 2).

In conclusion, the* CTLA4* genotype rs231775 AA may be one of risk factors for the development of malignancy in Chinese kidney transplant recipients. Besides other risk factors, rs231775 AA × primary diseases and rs231775 AA × number of HLA-mismatch became risk factors for incidence of tumor in the interaction (including gene-gene and gene-environment interaction). The haplotype TACAG was susceptible haplotype. Nevertheless, it would be necessary for our results to be confirmed in a larger study cohort.

## Supplementary Material

In the supplementary material, the Supplementary figure 1 showed association of the CTLA4 genotypes with time of discovery of tumor. Supplementary table 1 gave results on the test of Hardy-Weinberg equilibrium in 5 locus of CTLA4 SNP. Supplementary table 2 and Supplementary table 3 displayed the genotype and allele distribution of the CTLA4 polymorphisms in patients respectively. Supplementary table 4 illustrated association between tumor and several risk factors.

## Figures and Tables

**Table 1:** Comparison of clinical characteristics between patients with tumor and nontumor.

Characteristic	Patients with tumor (*n* = 53)	Patients with nontumor (*n* = 411)	*P* value
Mean age ± SD	40.717 ± 9.474	40.175 ± 10.100	0.712
Sex			
Male	35 (66.04)	253 (61.56)	0.552
Female	18 (33.96)	158 (38.44)	
Primary diseases			
Chronic glomerulonephritis	47 (88.68)	377 (91.73)	0.458
Polycystic kidney	1 (1.89)	17 (4.14)	0.426
Pyelonephritis	2 (3.77)	14 (3.40)	0.891
Aristolochic acid nephropathy	3 (5.66)	3 (0.73)	**0.003**
Dialysis time (≥3 years)	23	193	0.625
Number of HLA-mismatch	2.57 ± 1.029	2.47 ± 0.806	0.514
Real transplantation			
Living/cadaver	5/48	46/365	0.819
Antibody induction therapy	17	140	0.774
Immunosuppressant regimens			
CsA + MMF + Pred	34 (64.15)	272 (66.18)	0.760
TAC + MMF + Pred	19 (35.85)	139 (33.82)	
Blood transfusion	5	52	0.658
Rejection			
AR/non-AR	11/42	53/358	0.137

CsA: cyclosporine, MMF: mycophenolate mofetil, Pred: prednisone, TAC: tacrolimus, AR: acute rejection, non-AR: nonacute rejection, and tumor: drug induced liver injury.

**Table 2:** Multiplicative interaction between possible risk factors of malignancy.

Variables^*^	Age	Sex	Primary diseases	Dialysis time (≥3 years)	Number of HLA-mismatch	Antibody induction therapy
Sex	0.994	—	—	—	—	—
Primary diseases	0.053	0.766	—	—	—	—
Dialysis time (≥3 years)	0.612	0.560	**0.043**	—	—	—
Number of HLA-mismatch	0.966	0.886	0.815	0.055	—	—
Antibody induction therapy	**0.001**	0.838	**0.002**	0.275	0.281	—
Rejection	0.080	0.590	0.094	0.057	0.981	0.282

^*^The figures in the table were *P* value.

**Table 3:** The flounce on incidence of malignancy for *rs231775 AA* in gene-environment interaction and gene-gene interaction.

Variables	Tumor (*n* = 53)	Nontumor (*n* = 411)	*P*_interaction_
Mean age ± SD	40.72 ± 9.47	40.18 ± 10.10	0.712
Sex			
Male/female	35/18	253/158	0.306
Primary diseases			**0.021**
Dialysis time (≥3 years)	23	193	0.078
Number of HLA-mismatch	2.57 ± 1.029	2.47 ± 0.806	**0.006**
Antibody induction therapy	17	140	0.364
Rejection			
AR/non-AR	11/42	53/358	0.331
