# Supplementary material for: CTLA4 Polymorphisms and De Novo Malignancy Risk after Renal Transplantation in Chinese Recipients
Source: Biomed Res Int. 2015 Jan 15;2015:986780. doi: 10.1155/2015/986780 (PMC4312582; doi:10.1155/2015/986780)
Supplement: Supplementary file 1 — In the supplementary material, the Supplementary figure 1 showed association of the CTLA4 genotypes with time of discovery of tumor. Supplementary table 1 gave results on the test of Hardy-Weinberg equilibrium in 5 locus of CTLA4 SNP. Supplementary table 2 and Supplementary table 3 displayed the genotype and allele distribution of the CTLA4 polymorphisms in patients respectively. Supplementary table 4 illustrated association between tumor and several risk factors. [file 986780.f1.pdf]

Supplementary materials

Supplementary figure 1. Association of the *CTLA4* genotypes with time of discovery of tumor

Associations of the *CTLA4* SNPs with the time of discovery of tumor in patients were analyzed by the Kaplan-Meier test. In the Supplemental figure 1h, comparison between groups carrying with rs231775 AA and rs231775 AG+ GG was statistic, p = **0.014.**

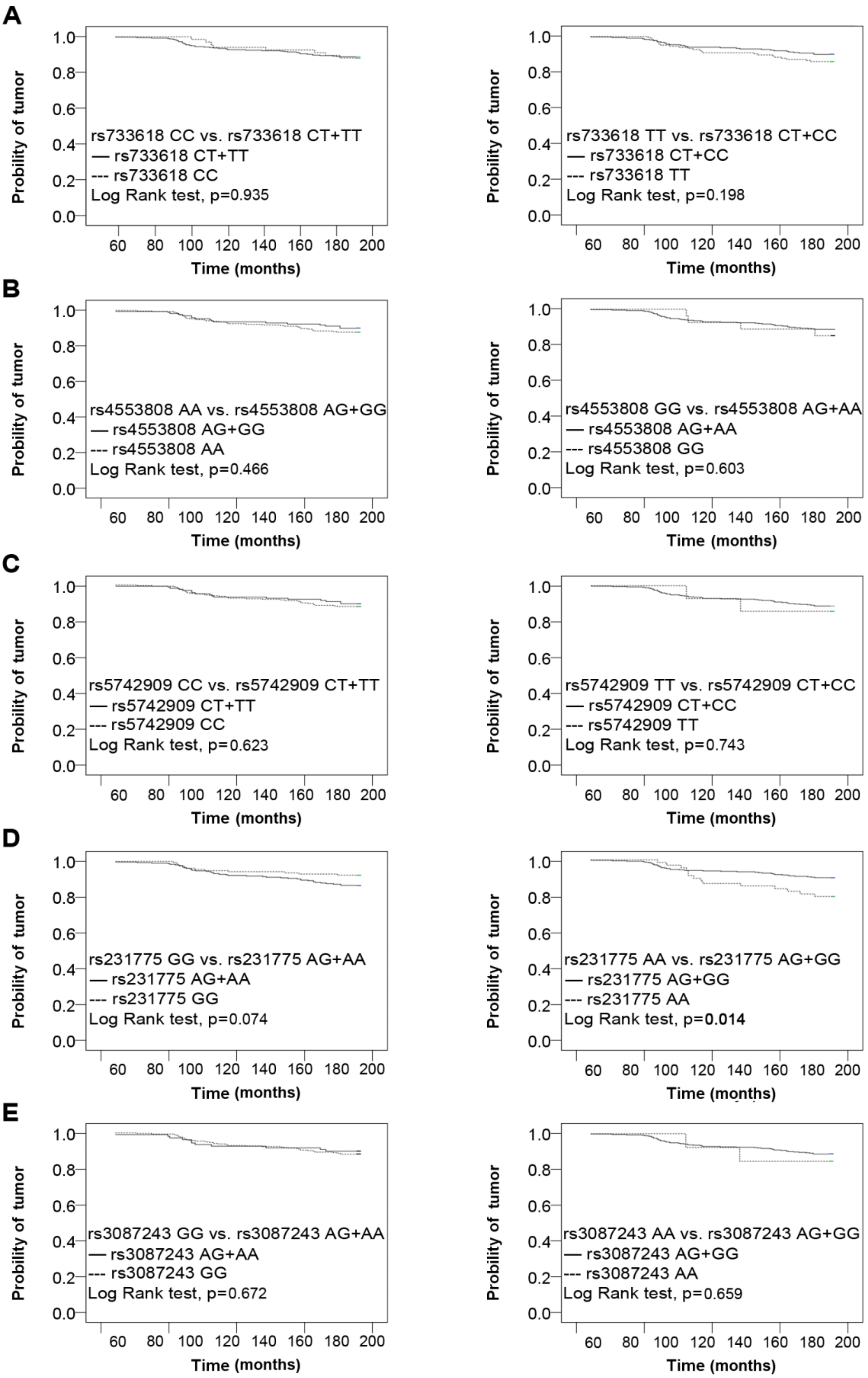
A
Probility of tumor
Time (months)
rs733618 CC vs. rs733618 CT+TT
— rs733618 CT+TT
--- rs733618 CC
Log Rank test, p=0.935
rs733618 TT vs. rs733618 CT+CC
— rs733618 CT+CC
--- rs733618 TT
Log Rank test, p=0.198
B
rs4553808 AA vs. rs4553808 AG+GG
— rs4553808 AG+GG
--- rs4553808 AA
Log Rank test, p=0.466
rs4553808 GG vs. rs4553808 AG+AA
— rs4553808 AG+AA
--- rs4553808 GG
Log Rank test, p=0.603
C
rs5742909 CC vs. rs5742909 CT+TT
— rs5742909 CT+TT
--- rs5742909 CC
Log Rank test, p=0.623
rs5742909 TT vs. rs5742909 CT+CC
— rs5742909 CT+CC
--- rs5742909 TT
Log Rank test, p=0.743
D
rs231775 GG vs. rs231775 AG+AA
— rs231775 AG+AA
--- rs231775 GG
Log Rank test, p=0.074
rs231775 AA vs. rs231775 AG+GG
— rs231775 AG+GG
--- rs231775 AA
Log Rank test, p=0.014
E
rs3087243 GG vs. rs3087243 AG+AA
— rs3087243 AG+AA
--- rs3087243 GG
Log Rank test, p=0.672
rs3087243 AA vs. rs3087243 AG+GG
— rs3087243 AG+GG
--- rs3087243 AA
Log Rank test, p=0.659

Supplementary table 1 Test of Hardy-Weinberg equilibrium in 5 locus of CTLA4 SNP

Locus	Genotype	Patients with tumor (n=53) n（%）		*p* value	Patients with non-tumor (n=411) n（%）		*p* value
rs733618	TT	23	0.504	0.477	141	1.975	0.159
	CT	22			211		
	CC	8			59		
rs4553808	AA	36	2.755	0.096	258	1.485	0.222
	AG	13			130		
	GG	4			23		
rs5742909	CC	36	0.076	0.781	265	1.023	0.311
	CT	15			134		
	TT	2			12		
rs231775	GG	12	0.021	0.882	145	2.531	0.111
	AG	27			211		
	AA	14			55		
rs3087243	GG	42	2.357	0.124	314	2.848	0.091
	AG	9			86		
	AA	2			11		

Supplementary table 2 The genotype distribution of the *CTLA4* polymorphisms in patients

Locus	*Genotype*	*Patients with tumor (n=53) n（%）*	*Patients with non-tumor (n=411) n（%）*	*Model*	*OR (95% CI)*	*p value*
rs733618	*TT*	*23 (43.40)*	*141 (34.31)*	*Dominant*	*1.061 (0.476-2.363)*	*0.837*
	CT	*22 (41.51)*	*211 (51.34)*	*Recessive*	*1.468 (0.822-2.622)*	*0.222*
	CC	*8 (15.09)*	*59 (14.35)*			
rs4553808	*AA*	*36 (67.92)*	*258 (62.77)*	*Dominant*	*1.377 (0.457-4.148)*	*0.568*
	AG	*13 (24.53)*	*130 (31.63)*	*Recessive*	*1.256 (0.682-2.312)*	*0.545*
	GG	*4 (7.55)*	*23 (5.60)*			
rs5742909	*CC*	*36 (67.92)*	*265 (64.48)*	*Dominant*	*1.304 (0.284-5.992)*	*0.732*
	CT	*15 (28.30)*	*134 (32.60)*	*Recessive*	*1.167 (0.633-2.150)*	*0.650*
	TT	*2 (3.77)*	*12 (2.92)*			
rs231775	*GG*	*12 (22.64)*	*145 (35.28)*	*Dominant*	*2.324*	***0.012****

					(1.185-4.556)	
	AG	*27 (50.94)*	*211 (51.34)*	*Recessive*	*0.537 (0.274-1.054)*	*0.089*
	AA	*14 (26.42)*	*55 (13.38)*			
rs3087243	*GG*	*42 (79.25)*	*314 (76.16)*	*Dominant*	*1.180 (0.585-2.380)*	*0.732*
	AG	*9 (16.99)*	*86 (20.92)*	*Recessive*	*1.426 (0.307-6.616)*	*1.426*
	AA	*2 (3.77)*	*11(2.92)*			

OR: odds ratio, CI: confidence intervals, *Bonferroni-adjusted ***p*** **=0.06**

Supplementary table 3 The allele distribution of *CTLA4* polymorphisms in patients

Locus	Allele	Patients with tumor (n= 106) n（%）	patients with non-tumor (n= 822) n（%）	OR (95% CI)	*p* value
rs733618	T	68(64.15)	493(59.98)	0.837 (0.550-1.275)	0.408
	C	38(35.85)	329(40.02)		
rs4553808	A	85(80.19)	646(78.59)	1.103 (0.665-1.829)	0.705
	G	21(19.81)	176(21.41)		
rs5742909	T	87(82.07)	664(80.78)	1.090 (0.644-1.843)	0.749
	C	19(17.93)	158(19.22)		
rs231775	G	51(48.11)	501(60.95)	0.594 (0.396-0.892)	**0.011***
	A	55(51.89)	321(39.05)		
rs3087243	G	93(87.74)	714(86.86)	1.082 (0.585-2.001)	0.801
	A	13(12.26)	108(13.14)		

OR: odds ratio, CI: confidence intervals

***** Bonferroni-adjusted ***p* =0.055**

Supplementary table 4 Association between tumor and several risk factors

Variables	Multivariable analysis		
	**tumor(n=53)**	**Non-tumor(n=411)**	***p* value**
Mean age±SD	40.72± 9.47	40.18± 10.10	0.712
Sex			
Male/Female	35/18	253/158	0.528
Primary diseases			
Chronic glomerulonephritis	47	377	0.458
Polycystic kidney	1	17	0.426
Pyelonephophritis	2	14	0.891
Aristolochic acid nephropathy	3	3	0.003
Dialysis time (≥3year)	23	193	0.625
Number of HLA -mismatch	2.57±1.029	2.47±0.806	0.429
Real transplantation			0.701
lived /cadaver	5/48	46/365	
Antibody induction therapy	17	140	0.774
Immunosuppressive regiment			0.770
CsA +MMF+Pred	34	272	
TAC + MMF+Pred	19	139	
Blood transfusion	5	52	0.503
Rejection			0.119
AR/ non-AR	11/42	53/358	

rs231775 SNPs			0.012
AA			
AG+ GG			
Time of discovery of tumor	-	-	0.000

CsA: cyclosporine, MMF: mycophenolate mofetil, Pred: prednisone, TAC: tacolimous,

AR: acute rejection, non-AR: non-acute rejection
